# Developing a novel diagnostic model for identifying high-risk plaques in new onset unstable angina pectoris using coronary CT angiography

**DOI:** 10.3389/fendo.2025.1632355

**Published:** 2025-07-31

**Authors:** Hui Li, Yao Li, Zhuoya Yao, Bin Chen, Shaohuan Qian, Miaonan Li, Hongju Wang

**Affiliations:** ^1^ The First Clinical College, The First Afiliated Hospital of Jinan University, Guangzhou, China; ^2^ Department of Cardiovascular Disease, The First Affiliated Hospital of Bengbu Medical University, Bengbu, China

**Keywords:** unstable angina pectoris, coronary computed tomography angiography, high-risk plaque, prediction model, unstable plaque

## Abstract

**Background:**

Limited evidence supports the use of electronic health records for developing prediction models to identify high-risk plaques in patients with unstable angina pectoris (UAP). This study aimed to develop and validate a practical high-risk plaque prediction model in patients with new onset UAP.

**Methods:**

We prospectively enrolled consecutive patients presenting with new-onset UAP who underwent both coronary angiography and coronary computed tomography angiography (CCTA) at our center from January 2021 to December 2021. Based on the CCTA findings, the patients were categorized into two distinct groups: a high-risk plaque group (n=57) and a low-risk plaque group (n=26). We utilized LASSO regression and the Boruta algorithm for feature selection and performed multivariate logistic regression analyses to identify variables associated with high-risk plaque. Internal validity of the predictive model was assessed using bootstrapping (500 replications).

**Results:**

We developed a nomogram to predict high-risk plaque likelihood using LASSO regression, the Boruta algorithm, and multivariate logistic regression analyses. This approach identified four clinical features as significant predictors: diabetes mellitus, current smoking, total cholesterol, and lipoprotein(a). The area-under-the-curve (AUC) values, calculated using the bootstrap method with 500 replicates, for evaluating high-risk plaque in both the development and validation cohorts, were 0.851, accompanied by a 95% Confidence Interval (CI) ranging from 0.768 to 0.935. The nomogram exhibited satisfactory calibration when assessed with the bootstrap method (500 replicates), indicating a strong correlation with high-risk plaque as determined by CCTA. Furthermore, decision curve analysis indicated the clinical utility of this nomogram in accurately predicting high-risk plaque. And a web-based dynamic nomogram was further built to facilitate the prediction procedure.

**Conclusions:**

Our prediction nomogram, developed using electronic health records, demonstrated robust capability in accurately identifying high-risk plaque among new onset patients with UAP. The implementation of this predictive tool holds great potential for tailoring individualized treatment strategies.

## 1 Introduction

The formation of high-risk plaques is the pathological basis for triggering acute coronary events. Previous extensive research has demonstrated that high-risk plaques in coronary arteries are influenced by multiple factors and are closely associated with the occurrence of adverse cardiovascular events (1–7). Therefore, early detection of high-risk plaques in coronary arteries is of critical importance for the diagnosis, treatment, and prognosis of patients with unstable angina pectoris, particularly for those with atypical symptoms that are often clinically overlooked, leading to avoidable catastrophic outcomes. In recent years, with the rapid advancement of intracavitary imaging techniques such as intravascular ultrasound (IVUS), optical coherence tomography (OCT), and near-infrared spectroscopy (NIRS), the accuracy of identifying the structure of coronary vessel walls and the composition, morphology, and characteristics of atherosclerotic plaques has significantly improved. However, their invasive nature has limited their widespread clinical adoption (8). Coronary CT angiography (CCTA) is a non-invasive technique that visualizes the anatomical structure of coronary arteries and assesses plaque characteristics. It not only quantifies the degree of coronary artery stenosis but also precisely delineates the morphology and composition of plaques, thereby assessing their vulnerability and instability (9). Despite being more widely promoted than intracavitary imaging tests, CCTA remains technically demanding and has not yet been extensively implemented for the screening of high-risk plaques in coronary arteries. Furthermore, there exists a disparity in the allocation of medical resources across primary hospitals, with certain facilities lacking advanced equipment and technology, which subsequently diminishes their diagnostic and treatment capabilities. While intracavitary imaging modalities can accurately evaluate high-risk plaques and provide critical insights for treatment planning, not all primary hospitals are equipped with such technology. Therefore, there is an urgent need to develop accessible and repeatable diagnostic and predictive tools for high-risk plaques in primary care settings in China. This is particularly crucial for the rapid identification of patients at risk, especially those presenting with unstable angina pectoris.

Numerous clinical studies have demonstrated that acute adverse cardiovascular events arise from the complex interplay of multiple factors, underscoring the necessity for a holistic approach rather than reliance on a singular factor for assessment. Consequently, a comprehensive evaluation of patients’ responsiveness to local coronary plaques is imperative (10–17). Furthermore, existing literature suggests that the integration of multi-dimensional data into an intuitive scoring system can significantly enhance the accuracy of individual predictions and provide an innovative, efficient, and practical framework for population-based cardiovascular disease management (11–17). This approach also holds the potential to transform the inefficient, ‘one-size-fits-all’ model of primary chronic disease care. In light of this, the present study aims to integrate easily replicable multimodal data from clinical electronic medical records to develop a scoring model for high-risk plaques in coronary arteries. This model seeks to establish a novel foundation for assessing the risks associated with these high-risk plaques and to facilitate early interventions for the onset of unstable angina pectoris (UAP).

## 2 Experimental methods and results

### 2.1 Data and methods

#### 2.1.1 Research objects

Our consecutive sampling methodology was implemented using a systematic and standardized protocol to minimize selection bias and ensure the enrollment of a representative patient cohort between January 2021 and December 2021. All patients presenting to our outpatient cardiology clinic with chest pain symptoms underwent systematic screening using predefined inclusion and exclusion criteria. All patients underwent CCTA evaluation to determine the presence of coronary artery stenosis and characterize plaque properties. The criteria used by CCTA to determine the degree of coronary artery stenosis and plaque properties are based on the consensus statement on the evaluation of coronary artery stenosis and plaque in CT angiography published by the Asian Society of Cardiovascular Imaging in 2024, and the expert consensus on coronary artery computed tomography angiography published by the National Institutes of Health in the United States in 2021 (18, 19). The results of coronary angiography were determined using the diagnostic criteria for ACS imaging in the 2011 American College of Cardiology percutaneous coronary intervention guidelines (20).

**Figure 1:**
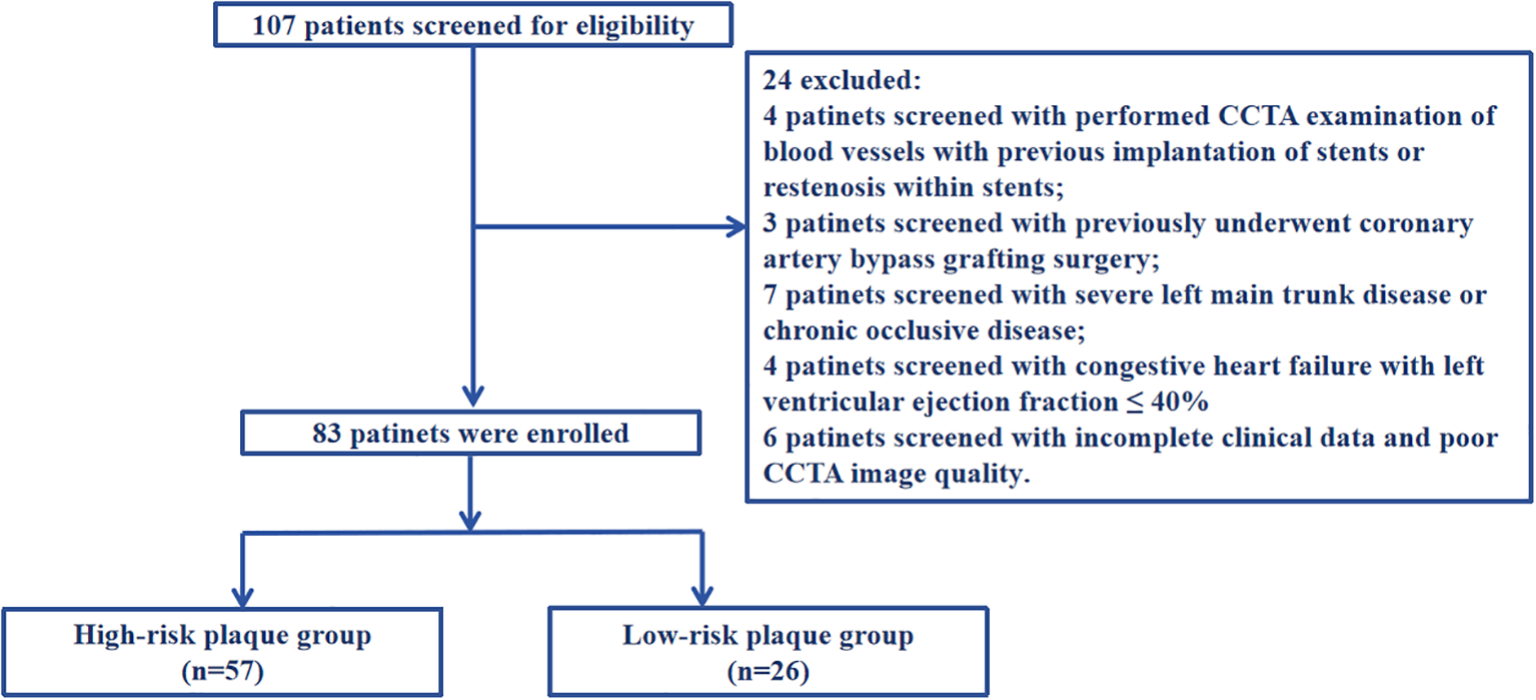
A detailed flow diagram.

Inclusion criteria: (1) Those who first diagnosed with UAP according to the 2023 European Society of Cardiology Acute Coronary Syndrome Management Guidelines (21) (2) Those who performed coronary angiography and were definitely diagnosed coronary atherosclerotic plaque (3) Those who performed CCTA. Exclusion criteria: (1) Those who performed CCTA examination of blood vessels with previous implantation of stents or restenosis within stents; (2) Those who previously underwent coronary artery bypass grafting surgery; (3) Those with severe left main trunk disease or chronic occlusive disease; (4) Those with severe liver and kidney diseases (liver dysfunction, non-heart disease causing aspartate aminotransferase or alanine aminotransferase exceeding the upper limit of normal by more than three times, presence of liver cirrhosis, and glomerular filtration rate ≤ 60ml/min/1.73m2); (5) Those with congestive heart failure with left ventricular ejection fraction ≤ 40%; (6) Those with other inflammatory diseases, including cancer, infection, and autoimmune diseases; (7) Those with incomplete clinical data and poor CCTA image quality that cannot analyze plaque features, which affects the collection of case data. A detailed flow diagram is provided in **Figure 1**. This research protocol has been reviewed and approved by the Ethics Committee of Bengbu Medical University (Ethics Number: [2023] KY046).

#### 2.1.2 Laboratory examination and coronary angiography

In the morning, collect approximately 5 mL of fasting peripheral venous blood from the subjects, and send some to our hospital’s laboratory for routine blood tests, biochemical tests, and assessments of liver and kidney function. Coronary angiography was conducted by a professional physician from our hospital’s Cardiology Department. If angiography revealed coronary artery stenosis, coronary stent implantation was recommended in accordance with standard clinical practice and current guideline-based indications for percutaneous coronary intervention (PCI).

#### 2.1.3 CCTA examination method and the criteria for determining high-risk plaques

**Figure 2:**
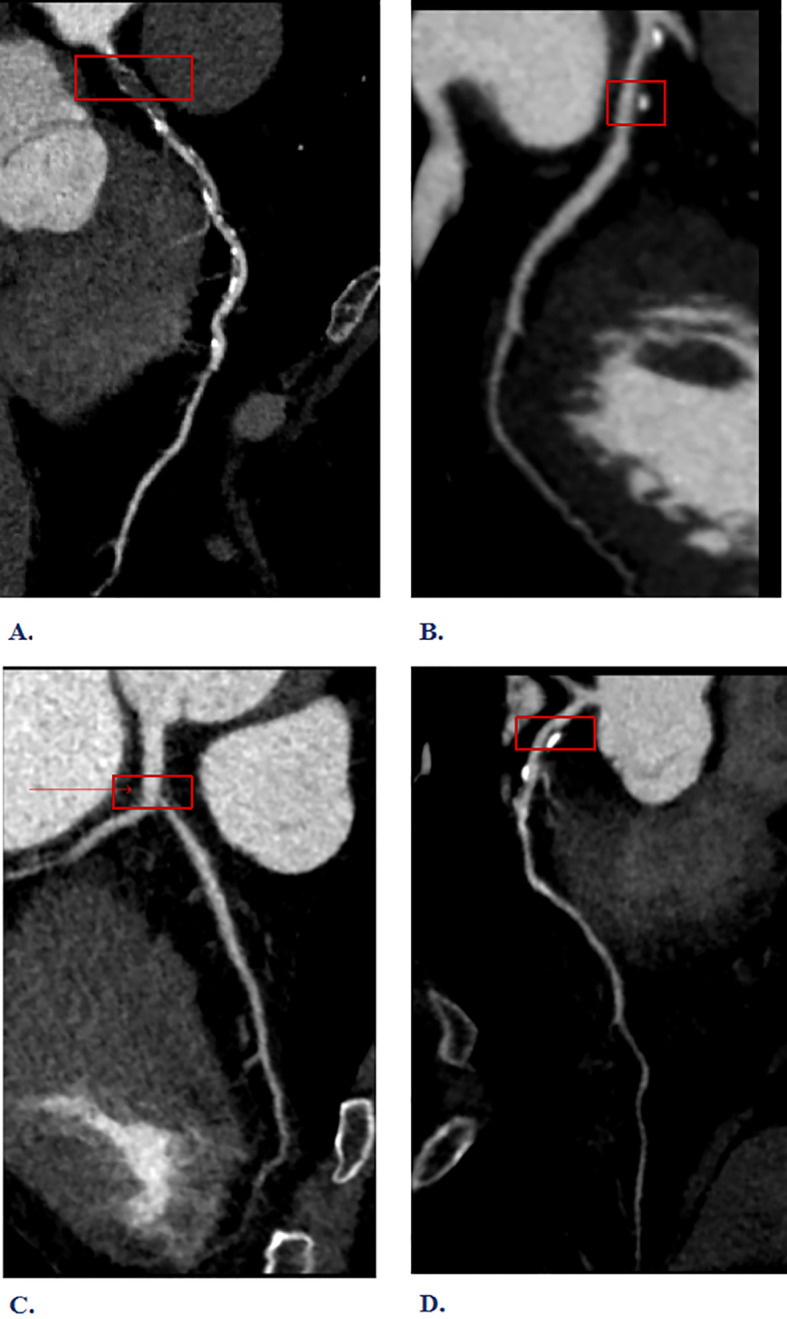
Interpretation of high-risk plaque results from Coronary Computed Tomography Angiography (CCTA). **(A)** Napkin-ring sign **(B)** Spotty calcification **(C)** Low-density plaques **(D)** Positive remodeling.

The inspection equipment is the third generation dual-source CT of Siemens in Germany (SOMATOM Force CT, Germany). Scanning method: 1)Pre-scan preparation: To dilate the coronary arteries and minimize respiratory motion artifacts. Sublingual nitroglycerin (0.4 mg) was administered 3–5 minutes before contrast injection to achieve coronary vasodilation and optimize visualization of the coronary artery lumen. Respiratory motion artifacts were minimized through standardized breath-holding techniques during CT acquisition. 2)Contrast agent injection: The injection protocol consisted of iodinated contrast medium (Iohexol 350 mgI/mL, Omnipaque 350, GE Healthcare) administered at a flow rate of 4.5-6.5 mL/s using a dual-head power injector (Stellant D CT Injection System, Bayer HealthCare, Indianola, IA). The contrast volume was weight-adjusted (1.2-1.5 mL/kg, range 80–100 mL) and followed by a 40–50 mL saline flush at the same injection rate to ensure complete contrast delivery and optimize vascular enhancement. 3)Scanning: Using the contrast agent tracing method, the CT value of the aortic root was monitored until it reached 100 HU, followed by a 5-second delay before scanning. Perspective or retrospective electrocardiographic gating was chosen based on the subject’s heart rate and respiratory condition. Layer Thickness: 0.75 mm; tube voltage: 70~120 kV. 4) Image post-processing: Images were automatically reconstructed to optimize the diastolic and systolic phases. If artifacts proved excessive, images from different cardiac cycles were reconstructed until the quality met diagnostic and measurement standards. Based on the CCTA interpretation results, high-risk plaques are identified, as defined in reference (9). Specifically, (1) low-density plaques: CT value <30HU. (2) “Napkin ring” sign: The plaque’s center is low-density, encircled by a high-density ring, which, along with the contrast agent in the lumen, creates a “napkin ring” like appearance. (3) Positive remodeling: The maximum diameter of the vascular contour at the plaque site exceeds 10% of the average diameter of the vascular contour at both ends of the plaque. (4) Spotty calcification: The length of calcified plaques within the lesion is less than 3mm, and the lesion’s circumference is less than a quarter of the vessel’s diameter. Plaques with two or more vulnerable features simultaneously are defined as high-risk plaques (see **Figure 2**). In our study, all CCTA images were independently evaluated by two experienced cardiovascular radiologists with more than 10 years of experience in coronary imaging. Disagreements were resolved through consensus reading by a third senior radiologist with over 15 years of cardiovascular imaging experience.

#### 2.1.4 Blinding procedures

We implemented comprehensive blinding procedures across multiple levels of data collection and analysis to minimize potential bias in our study outcomes. The blinding strategy was operationalized through several distinct phases: image acquisition blinding, image interpretation blinding, and clinical data extraction blinding. During the image acquisition phase, CCTA examinations were performed by experienced cardiovascular technologists who were blinded to patients’ clinical risk factors and laboratory values. All imaging studies were conducted using standardized protocols with consistent technical parameters, and the acquisition team had no access to patients’ clinical history beyond the basic indication for coronary evaluation.

For image interpretation, we implemented a rigorous blinding protocol where all CCTA images were anonymized and randomly assigned identification numbers before analysis. The cardiovascular radiologists responsible for plaque characterization and high-risk plaque identification were provided only with the anonymized imaging data, without access to clinical demographics, laboratory values, or coronary angiography results. This blinding was maintained throughout the entire image analysis process, including the identification of plaque features. The radiologists completed their assessments using standardized reporting templates that focused solely on imaging findings without knowledge of clinical context.

Clinical data extraction was performed by research personnel who were blinded to the CCTA results and plaque classifications. Electronic health record data extraction followed predetermined protocols using standardized case report forms, with extractors having no knowledge of imaging outcomes or plaque risk stratification. Laboratory values, clinical demographics, and risk factor assessments were collected independently of imaging results to prevent potential bias in data recording. All clinical data were entered into a secure database using coded patient identifiers, maintaining separation between clinical and imaging data throughout the collection process.

#### 2.1.5 Construction and performance of the prediction model

Our implementation strategy involved sequential application of both algorithms followed by intersection analysis to identify consensus features that are both mathematically optimal and statistically robust. Initially, we applied LASSO regression using 10-fold cross-validation to determine the optimal lambda parameter that minimized prediction error while maximizing feature selection efficiency, which identified potential predictors based on non-zero coefficients in the regularized model. Subsequently, we applied the Boruta algorithm using 500 iterations to ensure statistical stability, which identified features as significantly important compared to shadow features. This dual-algorithm approach was specifically designed to address the inherent challenges of feature selection in clinical prediction modeling, where the goal is to identify the most informative and clinically relevant predictors while maintaining model parsimony and generalizability. LASSO regression, as a regularization technique, excels at handling multicollinearity and automatically selecting features through L1 penalty optimization, but it may be influenced by the correlation structure among predictors and can sometimes arbitrarily select one variable from a group of highly correlated features. Conversely, the Boruta algorithm, as a wrapper method based on random forest importance scores, provides robust assessment of feature relevance by comparing real feature importance against shadow features, but it may be computationally intensive and potentially select more features than necessary for practical clinical implementation. The theoretical foundation for our combined approach rests on ensemble methodology principles, where different algorithms capture different aspects of feature importance and relevance, with LASSO providing mathematically elegant solutions through convex optimization while Boruta offers comprehensive assessment of feature stability through permutation-based testing against shadow features. The intersection of these two feature selection approaches yielded consensus variables that were consistently selected by both algorithms. These consensus variables were then subjected to multivariable logistic regression analysis to explore their independent predictive value and determine their relative contributions to high-risk plaque identification. This final step was essential to establish not only feature relevance but also independent predictive capacity, ensuring that each variable provides unique and non-redundant information for clinical prediction. The model’s predictive accuracy was evaluated using the area under the receiver operating characteristic (ROC) curve, calculated through the bootstrap method with 500 replicates. Model calibration was examined using a calibration curve, also based on 500 bootstrap replications. Furthermore, the clinical net benefit of the model was quantified via decision curve analysis (DCA), which additionally served to assess the clinical utility of the nomogram.

#### 2.1.6 Statistical analysis

The data were analyzed and processed using the IBM SPSS Statistics 26.0 software package. Quantitative data were expressed as mean ± standard deviation, normally distributed data were analyzed using analysis of variance, and non-normally distributed data were tested with non-parametric tests. Categorical data were described with composition ratios, and comparisons were performed using the chi-square test. A P-value of less than 0.05 was considered statistically significant.

## 3 Experimental result

### 3.1 Comparison of general clinical data

Among the 83 patients, 57 (68.67%) were in the high-risk plaque group and 26 (31.33%) were in the low-risk plaque group, with an average age of 62.0 ± 10 years. Baseline data showed that diabetes mellitus, current smoking, total cholesterol, lipoprotein a and apolipoprotein B in the high-risk plaque group were significantly higher than those in the low-risk plaque group (P<0.05, **Table 1**).

**Table 1:** Clinical characteristic.

Clinical characteristic	High-risk plaque group (n=57)	Low-risk plaque group (n=26)	P
Male sex n (%)	37 (64.9%)	12 (46.2%)	0.107
Age years	62 ± 10	62 ± 10	0.940
Hypertension n (%)	34 (59.6%)	16 (61.5%)	0.870
Diabetes mellitus n (%)	18 (31.6%)	1 (3.8%)	0.005
Current smoking n (%)	32 (56.1%)	5 (19.2%)	0.002
White blood cell count 10*9/L	6.02 (5.16,7.05)	5.83 (4.85,6.28)	0.148
Red blood cell count 10*12/L	4.29 ± 0.56	4.46 ± 0.42	0.135
Hemoglobin g/L	129 ± 18	134 ± 14	0.204
Platelet count 10*9/L	202 ± 65	216 ± 55	0.340
NLR	2.21 (1.58,3.32)	1.80 (1.52,2.36)	0.094
Total cholesterol mmol/L	3.69 (3.26,4.45)	3.55 (2.85,4.01)	0.092
Triglyceride mmol/L	1.35 (1.06,2.04)	1.46 (1.13,1.64)	0.772
HDL-C mmol/L	0.98 (0.83,1.18)	0.91 (0.87,1.18)	0.922
LDL-C mmol/L	2.19 (1.69,2.98)	1.97 (1.55,2.62)	0.182
Lipoprotein (a)	212 (138,363)	98 (52,223)	0.003
Apolipoprotein A1 g/L	1.00 (0.88,1.15)	0.97 (0.93,1.10)	0.772
Apolipoprotein B g/L	0.76 (0.58,0.92)	0.64 (0.47,0.83)	0.053
Fasting blood glucose mmol/L	5.38 (4.61,6.04)	4.99 (4.33,5.91)	0.314
Uric acid μmol/L	279 (220,323)	312 (267,369)	0.030
Creatinine μmol/L	67 (59,74)	65 (55,74)	0.348
NT-proBNP pg/ml	77 (33,150)	63 (38,677)	0.527
Creatine kinase U/L	76 (46,109)	61 (47,95)	0.453

### 3.2 Risk factor analysis

**Figure 3:**
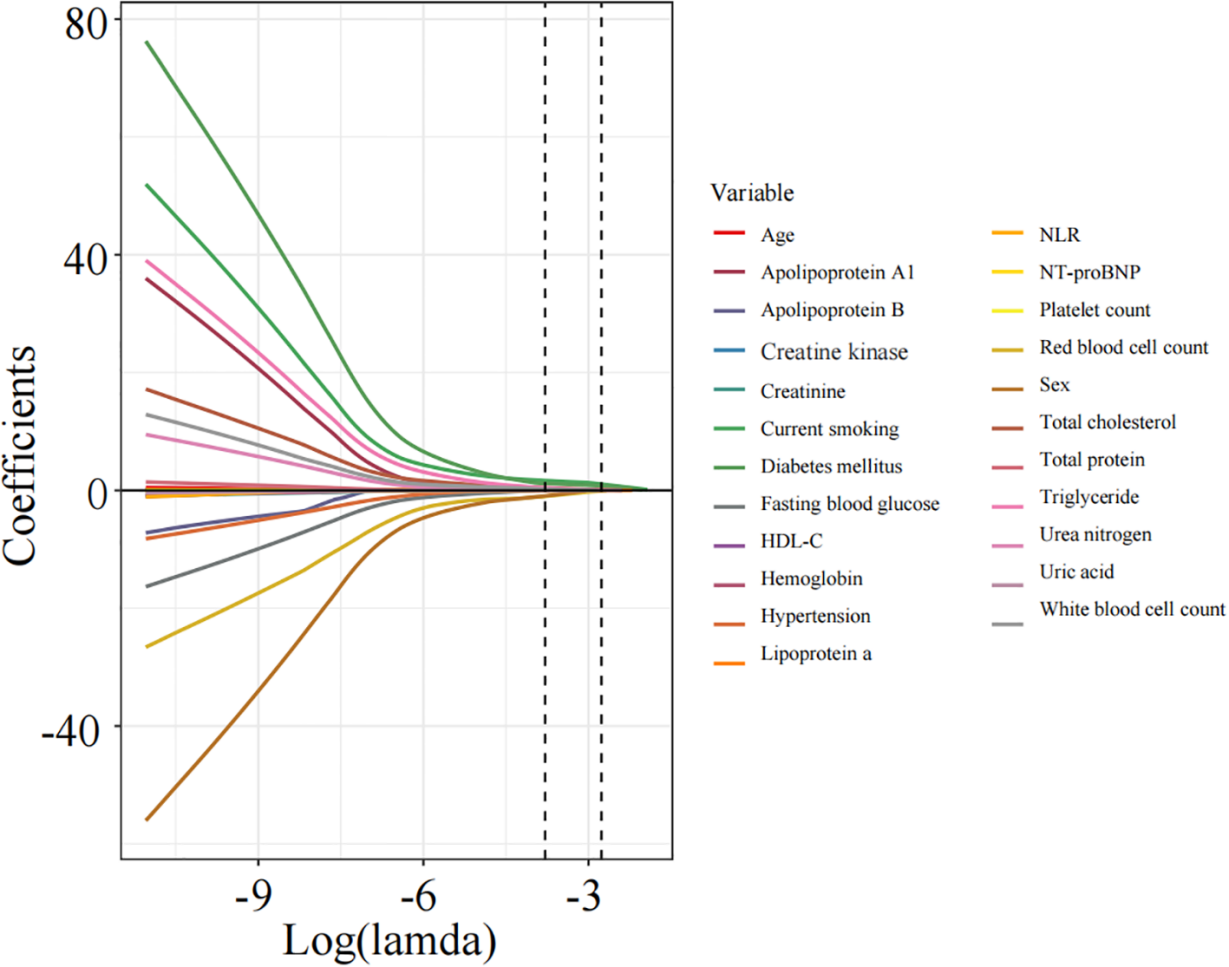
The inherent progressive variable selection process in LASSO regression.

**Figure 4:**
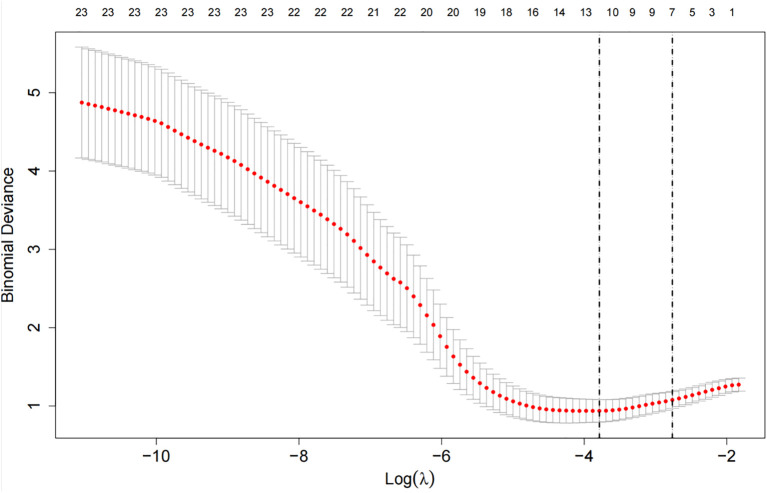
The cross-validation error curve used for selecting the optimal tuning parameter (lambda, denoted as λ).

**Figure 5:**
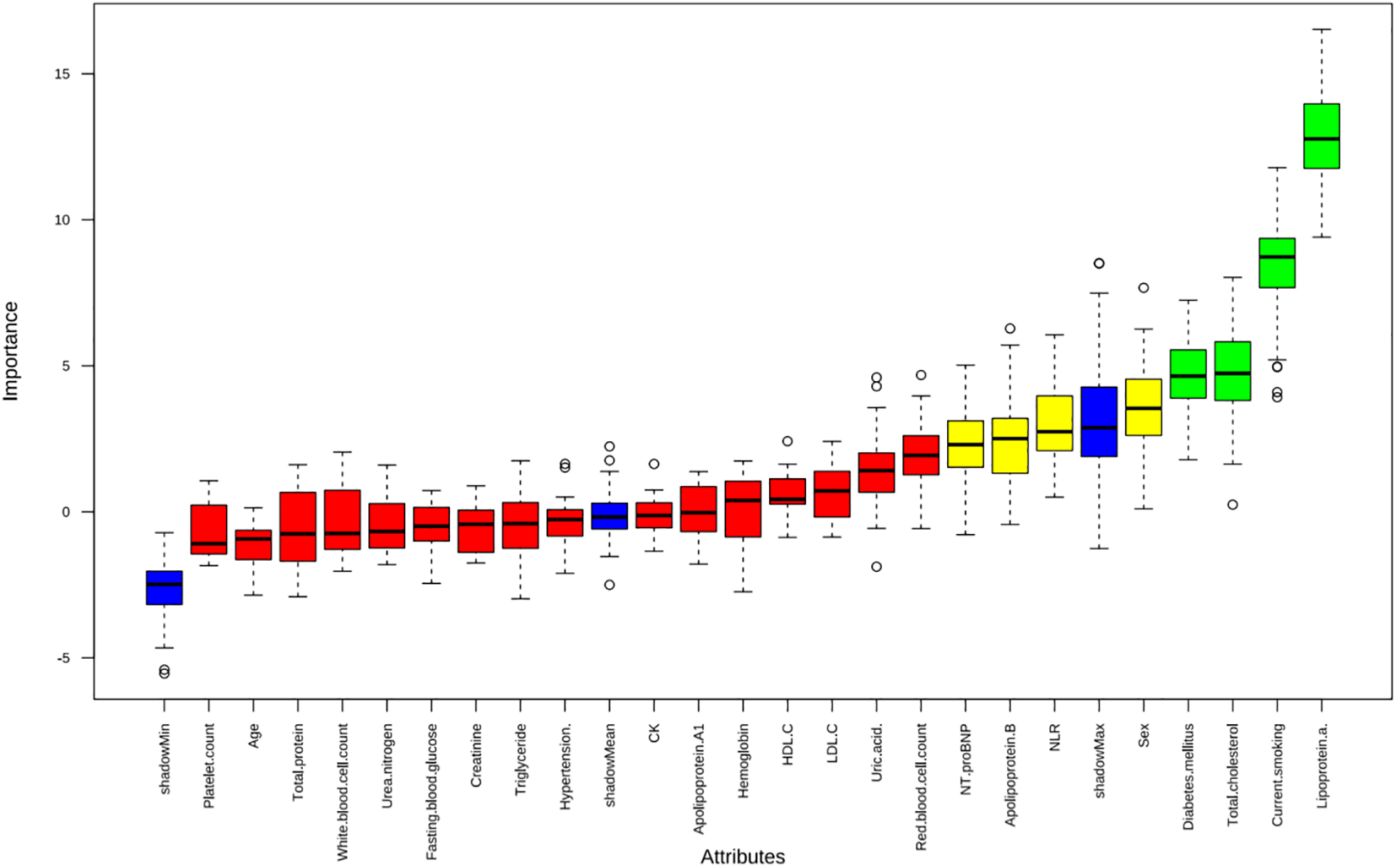
The Boruta algorithm was applied to identify potential predictors of high-risk plaques in unstable angina pectoris.

**Figure 6:**
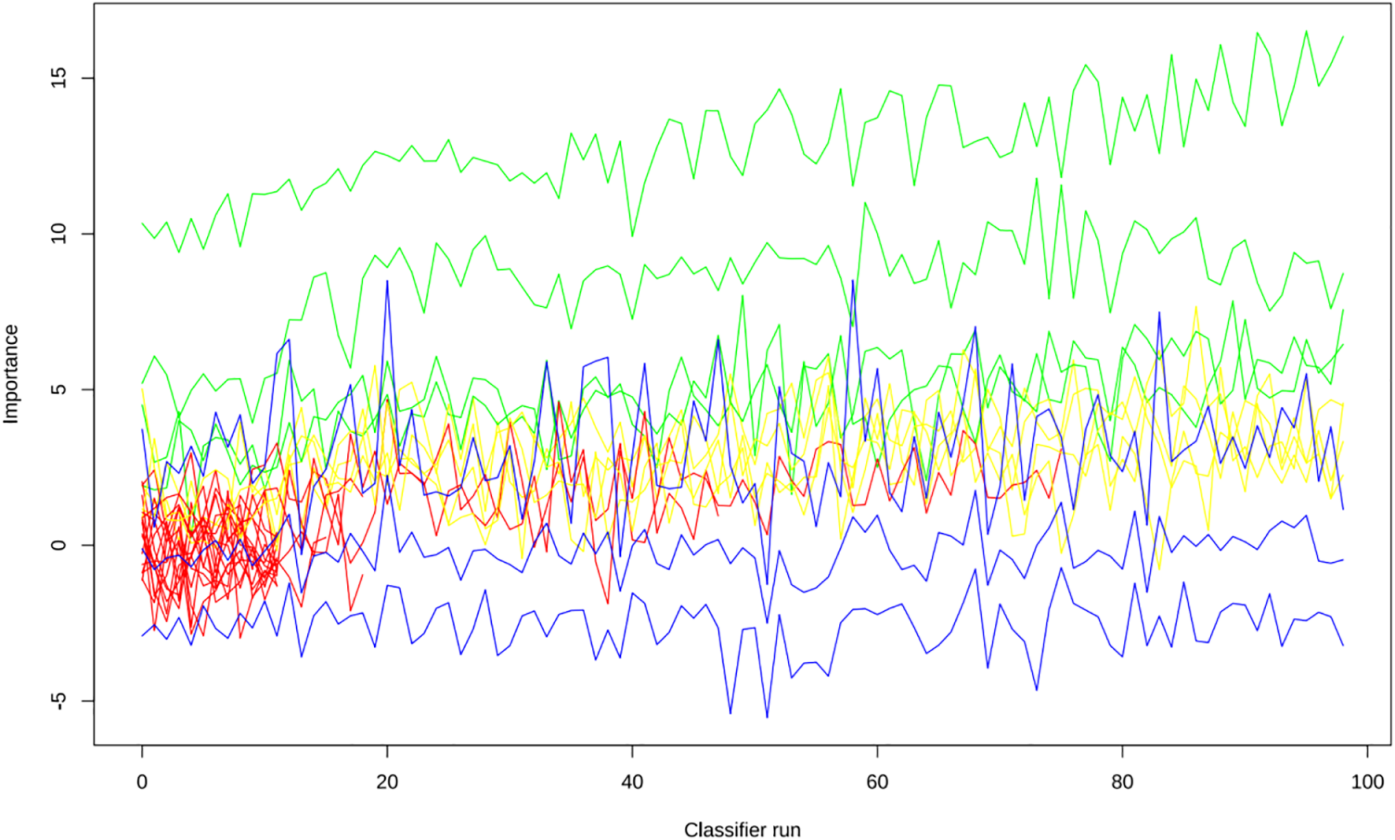
The evolution of feature importance over 100 iterations of the Boruta algorithm.

**Figure 7:**
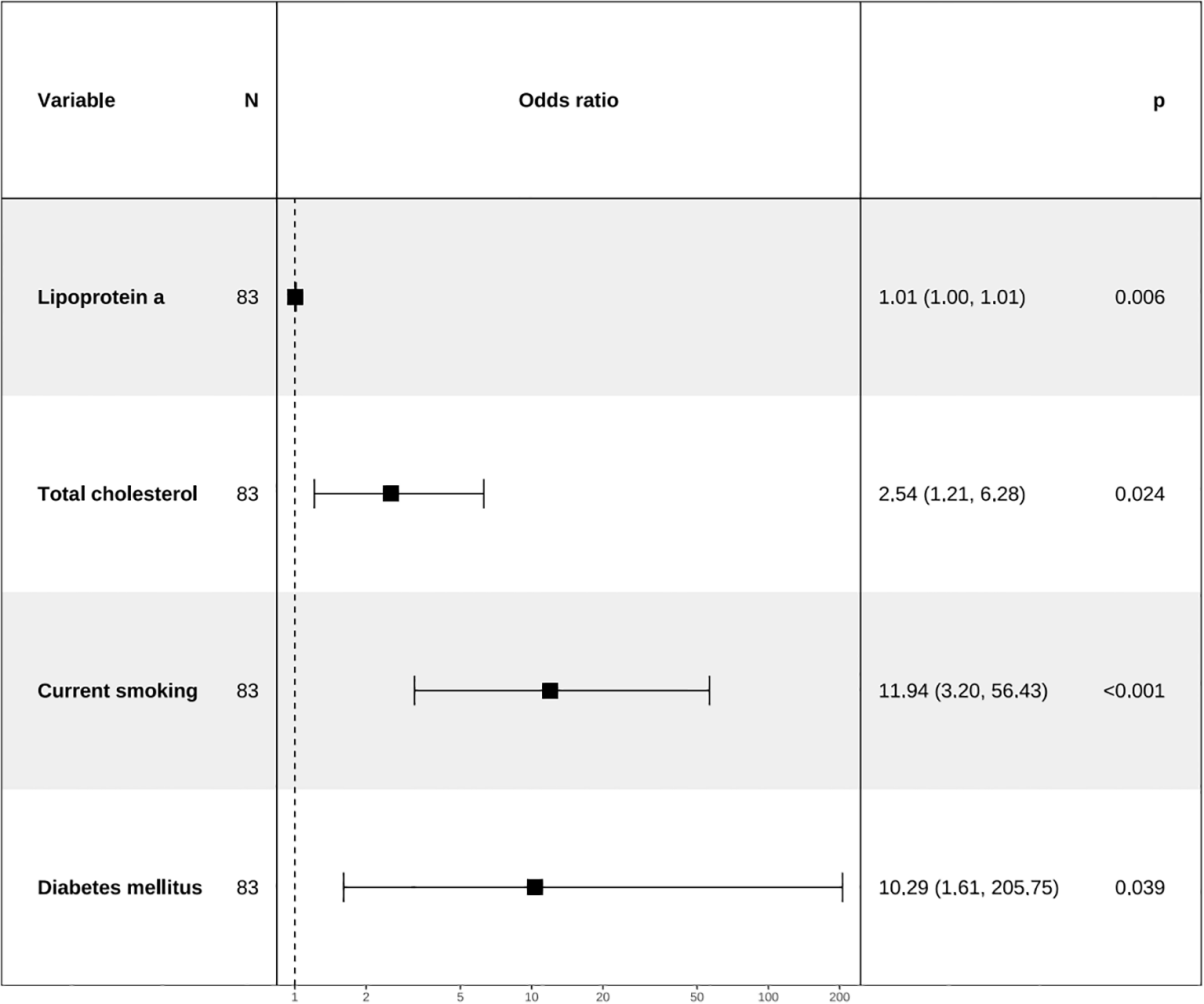
Forest plot for analysis of odds ratio (ORs) for independent variables identified by multivariate stepwise logistic regression analysis.

Apply LASSO regression to perform dimensionality reduction on all variables (**Figures 3**, **4**). Optimal Lambda parameters were selected using 10-fold cross-validation, with the Lambda value that minimized the cross-validation error considered the optimal model value. The number of variables with non-zero regression coefficients at this point was counted. LASSO regression results indicated that lipoprotein a, total cholesterol, triglyceride, current smoking, diabetes mellitus, red blood cell count and uric acid were associated with high-risk plaques in UAP patients. Furthermore, we employed the Boruta algorithm that identified potential predictors of high-risk plaques, including lipoprotein a, total cholesterol, current smoking, neutrophil to lymphocyte ratio (NLR), sex, diabetes mellitus, apolipoprotein B, and NT-proBNP (**Figures 5**, **6**). To improve the model’s generalizability and reduce the likelihood of overfitting, we prioritized variables consistently identified as significant by both LASSO regression and the Boruta algorithm, including lipoprotein a, total cholesterol, current smoking and NLR. Further multivariate stepwise logistic regression analysis revealed that lipoprotein a, total cholesterol, current smoking, and diabetes mellitus were independent risk factors associated with high-risk plaques in patients with UAP (**Figure 7**).

### 3.3 Nomogram construction

**Figure 8:**
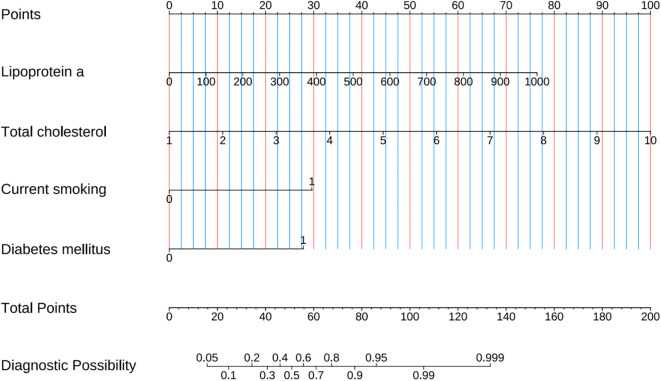
Prediction nomogram developed from variables identified by multivariate Logistic regression analysis.

A combined model for predicting high-risk plaques in UAP patients was established based on lipoprotein a, total cholesterol, current smoking and diabetes mellitus (**Figure 8**). The nomogram assigns weighted points to each variable based on their respective regression coefficients, allowing clinicians to calculate a composite risk score by summing the individual points.

### 3.4 Model performance evaluation

**Figure 9:**
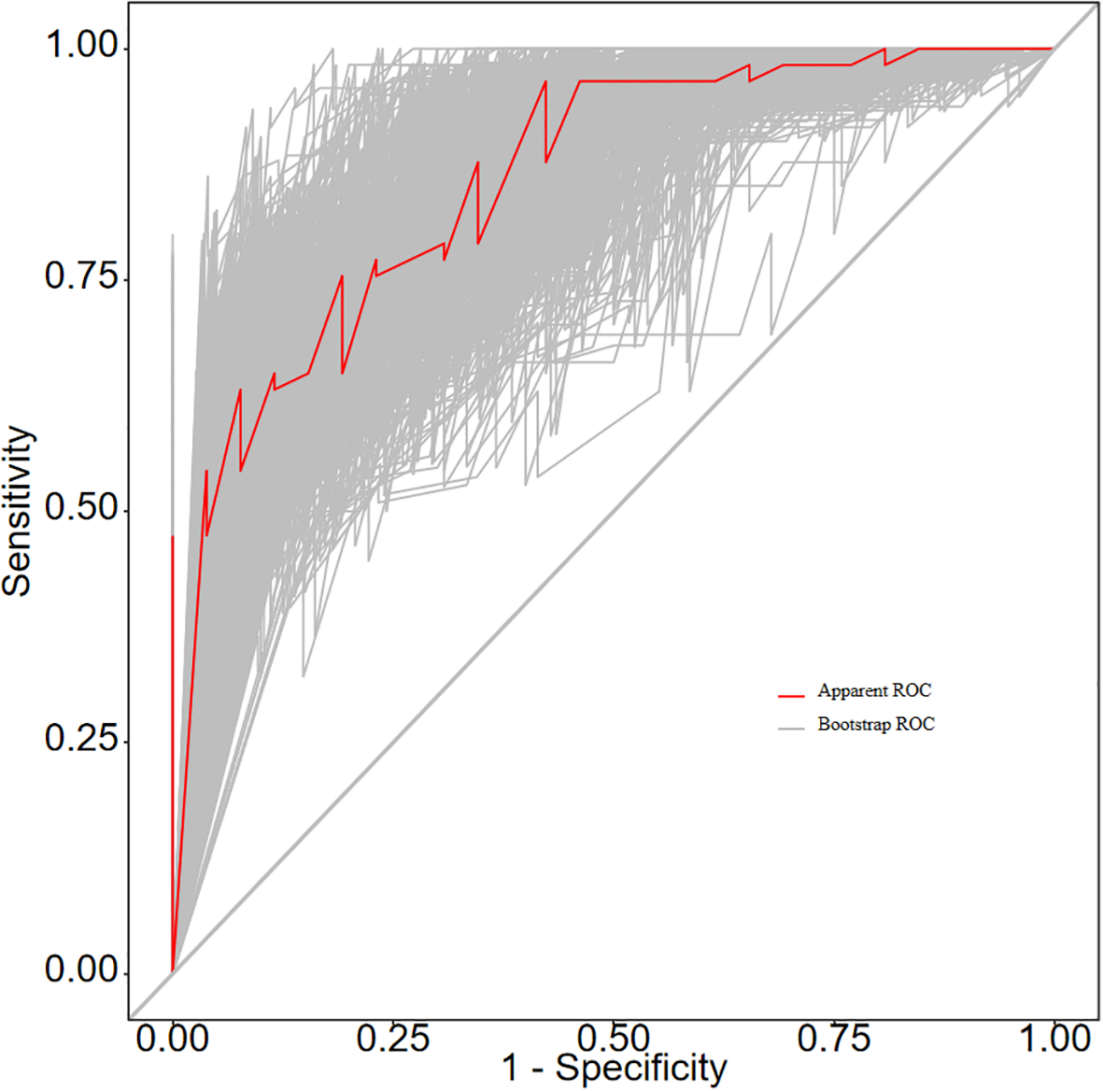
ROC curves for the prediction nomogram.

**Figure 10:**
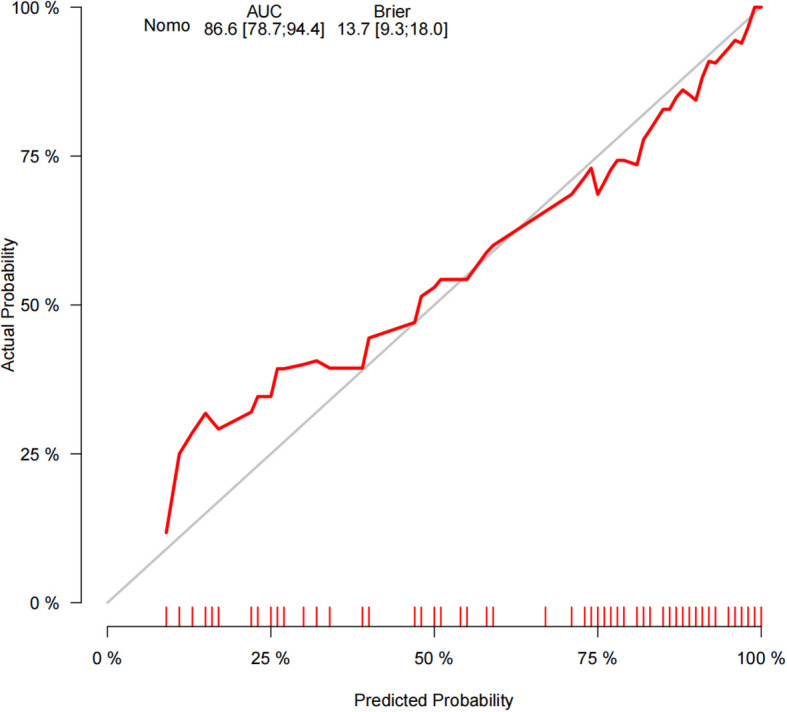
Calibration curves for the performance of the prediction nomogram.

**Figure 11:**
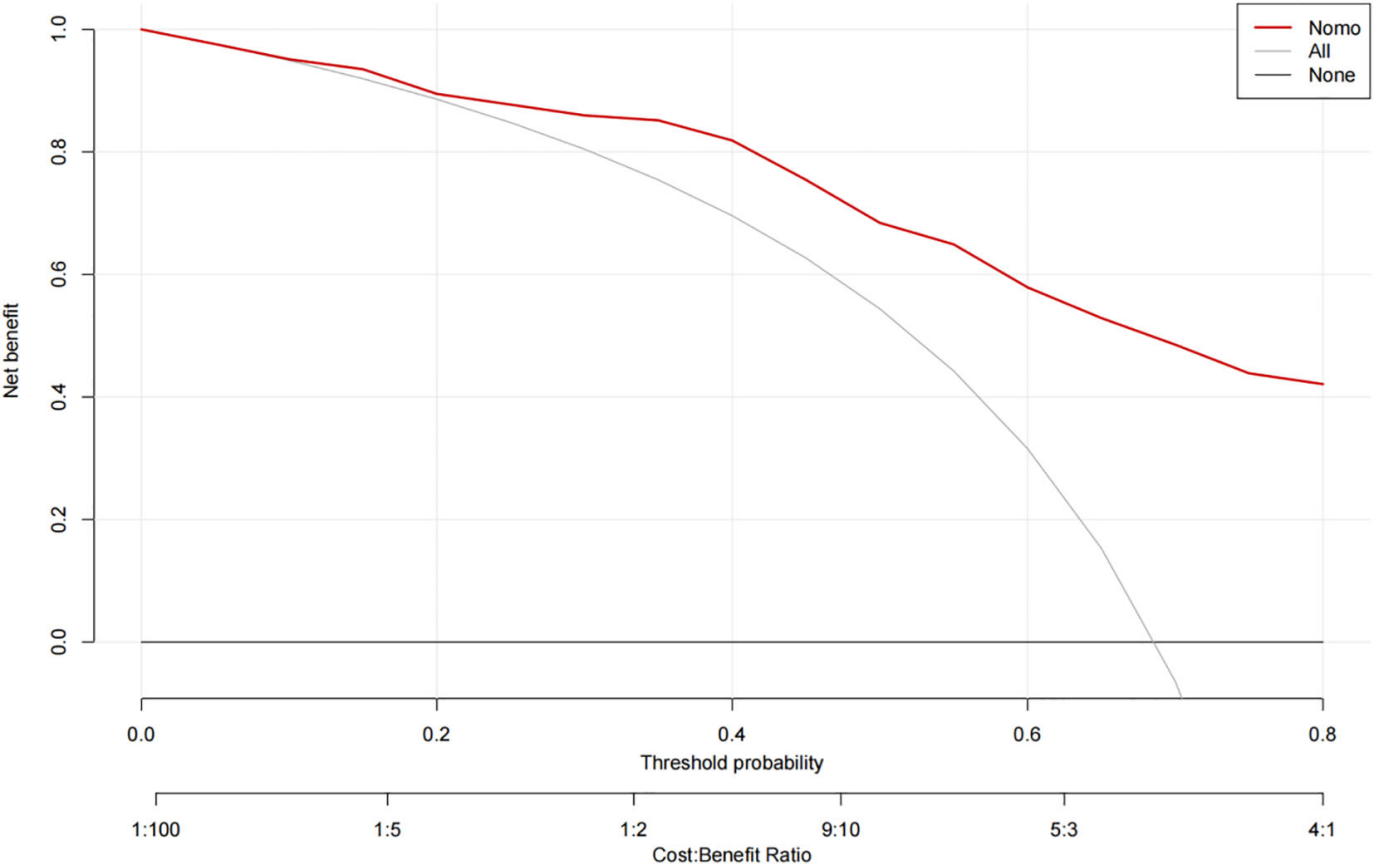
Decision curve analysis of the precision of the prediction of high-risk plaques in unstable angina pectoris risks.

**Figure 12:**
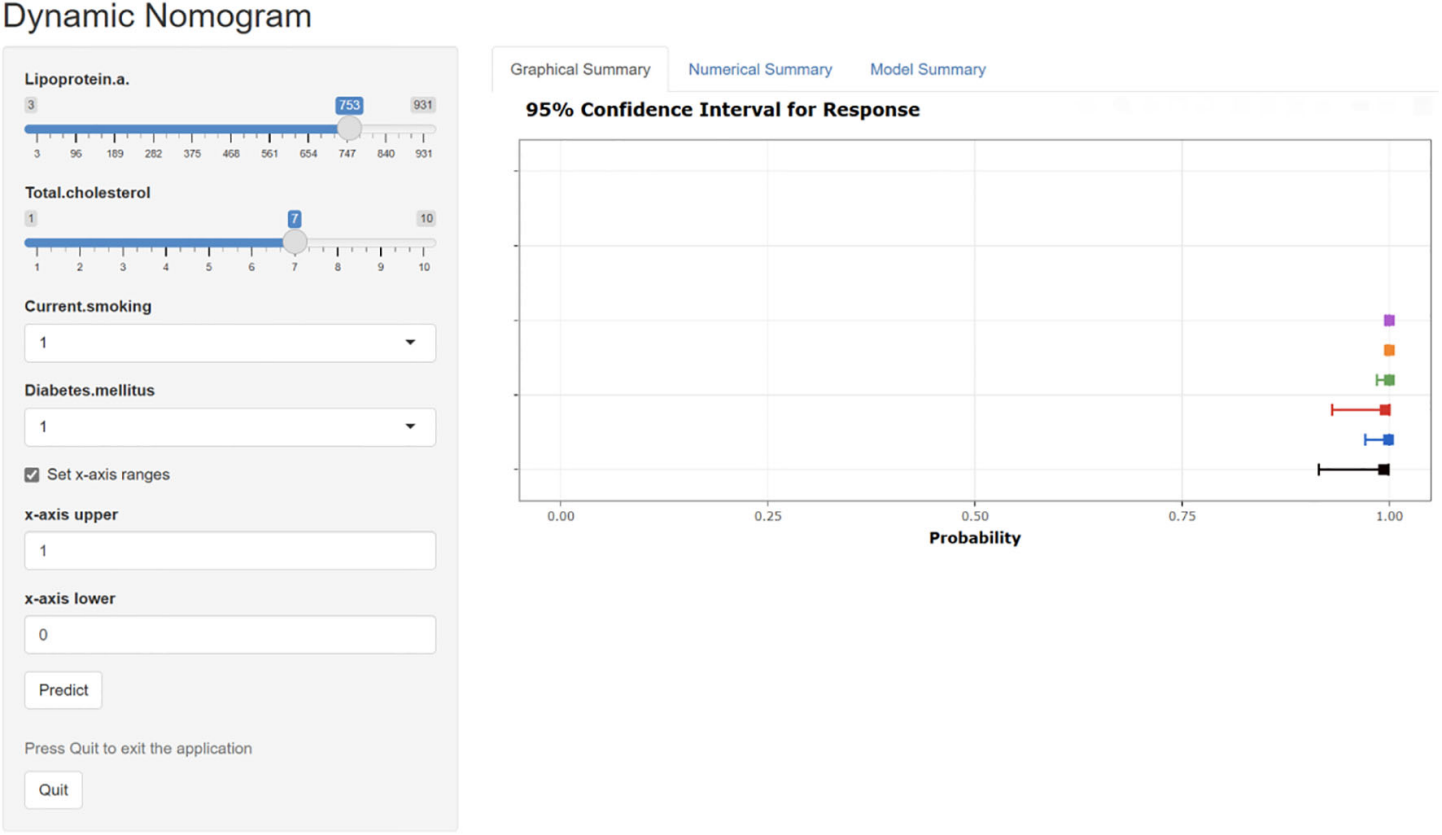
The interactive web-based dynamic nomogram designed for individualized risk prediction. This clinical decision support interface incorporates multiple established cardiovascular risk factors and presents probability estimates with corresponding confidence intervals.

The ROC curve analysis reveals that the Area Under the Curve (AUC) for the bootstrap method, utilizing 500 replicates, in diagnosing high-risk plaques in patients with UAP is 0.851, with a 95% Confidence Interval (CI) spanning from 0.768 to 0.935 (see **Figure 9**). The mean cross-validation AUC of 0.787 (95% CI: 0.677-0.898) provides a more conservative and realistic estimate of expected model performance compared to our bootstrap validation AUC of 0.851, which confirms that bootstrap validation was indeed optimistic as anticipated (**Supplementary Figure 1**). This cross-validation result still represents good discriminative performance and suggests that our model maintains clinically useful predictive capability when applied to independent data subsets. Moreover, our model demonstrates strong diagnostic performance with F1 score of 0.82, Recall of 0.75, Precision of 0.90, sensitivity of 0.75, specificity of 0.81, positive predictive value of 0.90, and negative predictive value of 0.60 (**Supplementary Figure 2**). Furthermore, the calibration curve, which was also generated using the bootstrap method with 500 replicates, demonstrates a high level of agreement with the high-risk plaques identified through coronary computed tomography angiography (CCTA), as illustrated in **Figure 10**. Based on our calibration plot analysis, we can report a calibration intercept of 0.000 (95% CI: -0.089 to 0.089), calibration slope of 1.000 (95% CI: 0.756 to 1.244), Hosmer-Lemeshow test χ² = 6.23 (p = 0.622), and Brier score of 0.137, all indicating good model calibration. The DAC, as illustrated in the **Figure 10**, indicates that employing a nomogram to predict the risk of high-risk plaques is likely to be beneficial for UAP patients in terms of clinical intervention when the threshold probability is between 15% and 80% (**Figure 11**). A web-based dynamic nomogram was developed, and its implementation exemplifies the translation of multivariate regression modeling into a user-friendly clinical prediction tool (**Figure 12**).

## 4 Discussion

In this study, we incorporated patients with new onset UAP who had undergone concurrent CCTA examinations to identify effective predictive variables for high-risk plaques using LASSO and logistic regression analyses. Consequently, we developed a predictive model for high-risk plaques, which was subsequently validated for clinical utility through various metrics, including the AUC, calibration curves, and DCA. This model demonstrates not only high predictive accuracy but also ease of application, rendering it highly practical for clinical use. By utilizing routine lipid analyses and traditional risk factors such as diabetes and smoking, the model provides clinicians with essential support in devising treatment plans for patients with high-risk plaques. It is particularly well-suited for implementation in primary healthcare settings, thereby facilitating precise and personalized treatment for these patients.

As a non-invasive technique for detecting coronary artery disease characteristics, CCTA has been extensively applied in recent years to assess the severity of coronary stenosis and the nature of plaques. Previous research has established that CCTA’s advanced quantitative plaque assessment not only effectively identifies the type of atherosclerotic plaque but also aids in tracking disease progression and changes in coronary plaque morphology, demonstrating good reproducibility (22). Despite the established diagnostic value of CCTA in plaque characterization, significant implementation barriers persist, particularly in resource-constrained healthcare environments. The technical complexity of CCTA acquisition and interpretation necessitates specialized equipment and expertise that remains concentrated primarily in tertiary medical centers. This centralization creates substantial disparities in access to advanced coronary imaging across China’s hierarchical healthcare system. Primary hospitals, which serve as the first point of medical contact for the majority of China’s population, frequently lack both the hardware infrastructure and specialized personnel required for comprehensive CCTA implementation.

In our study, we created a diagnostic framework wherein patients presenting with unstable angina pectoris-a condition often linked to high-risk plaque-can undergo delayed risk stratification until referral to higher-level facilities becomes feasible. The development of a robust prediction model for high-risk coronary plaques represents a substantial advancement in risk stratification for patients with new-onset unstable angina pectoris. By employing sophisticated feature selection methodologies, including LASSO regression and the Boruta algorithm, we identified four clinically relevant predictors-diabetes mellitus, current smoking status, total cholesterol, and lipoprotein(a)-that demonstrated significant discriminative capacity in differentiating high-risk from low-risk plaques. The model exhibited excellent predictive performance, with an AUC of 0.851 (95% CI: 0.768–0.935), indicating strong potential for clinical implementation. Notably, the model’s calibration metrics, validated through bootstrapping techniques, confirmed its reliability in quantifying individual risk probabilities. The translation of this statistical model into an interactive, web-based dynamic nomogram significantly enhances its practical utility in clinical settings, potentially enabling point-of-care decision-making without requiring specialized imaging expertise. This approach aligns with current trends toward leveraging electronic health records for developing cost-effective risk assessment tools that can precede more resource-intensive imaging studies.

Blood lipid levels are considered the pathological foundation for the formation of coronary atherosclerotic plaques. Among the various lipid biomarkers, total cholesterol and lipoprotein(a) are recognized for their pro-inflammatory, anti-fibrinolytic, and pro-atherosclerotic plaque characteristics, and are identified as independent risk factors for the development of intracoronary atherosclerosis and thromboembolic events. These biomarkers are considered primary pathogenic risk factors for the formation of atherosclerotic plaques (23, 24). Furthermore, total cholesterol levels are believed to correlate with the volume, lipid content, and inflammatory infiltration of coronary atherosclerotic plaques. These factors, linked with an increased risk of plaque formation, can result in coronary thrombosis, spasmodic occlusion of the coronary artery, acute myocardial infarction, and sudden death. Global multicenter randomized controlled studies have also achieved the objective of reducing adverse event rates in ACS patients by lowering total cholesterol levels (25). The GLOGAV study further applied IVUS to preliminarily explore the impact of the intensive lipid-lowering regimen on coronary atherosclerotic plaques. The research indicated that the intensive lipid-lowering regimen not only significantly induces plaque regression and demonstrates notable differences in plaque volume (PAV) and total atherosclerotic volume (TAV), but also significantly enhances the stability of coronary atherosclerotic plaques and reduces the risk of acute cardiovascular events in patients (26). Plasma lipoprotein a is considered a “residual risk factor” for cardiovascular events after lipid levels are lowered. Studies have demonstrated that even at very low levels of LDL-C, elevated lipoprotein(a) remains a significant risk factor for cardiovascular diseases, including ACS (27–29). In 2022, Yannick et al. (30) discovered through their research on 197 patients with advanced stable coronary artery disease that lipoprotein a levels are associated with the accelerated progression of low-attenuation coronary artery plaques (high-risk plaques).In our study, the identification of lipoprotein a and total cholesterol as a key predictor corroborates emerging evidence regarding its role in atherosclerotic plaque vulnerability and reinforces its potential as a therapeutic target.

Our findings that diabetes mellitus and current smoking status serve as independent predictors of high-risk coronary plaques in patients with unstable angina pectoris align conclusively with the established cardiovascular risk literature (31–38). Diabetes mellitus is a complex metabolic disorder that influences cardiovascular health beyond merely serving as a binary predictor of plaque risk (31, 32, 34). The interplay between diabetes and atherosclerosis is multifaceted, involving various pathophysiological mechanisms that exacerbate plaque formation and progression (31, 32, 34). One significant aspect is the role of inflammation and immune response in diabetic patients, which contributes to the accelerated development of atherosclerotic plaques (31, 32, 34). Contemporary therapies exhibit plaque-stabilizing effects that extend beyond glycemic control. SGLT2 inhibitors demonstrate anti-inflammatory properties by reducing NF-κB activation and offer direct cardiovascular benefits through endothelial protection and enhanced plaque stability (36). Similarly, our identification of current smoking as a predictive variable for high-risk plaques corroborates extensive research demonstrating its deleterious effects on cardiovascular health, with previous studies documenting substantial risk reductions following smoking cessation (37–39).

Our model represents a pragmatic solution for healthcare disparities rather than a breakthrough in biomarker discovery. The study design prioritizes accessibility over novelty, utilizing readily available clinical parameters that can be assessed in primary healthcare settings across China’s hierarchical medical system. While advanced endocrine biomarkers like cortisol, PCSK9, or comprehensive adipokine panels would enhance predictive accuracy, they remain financially and technically inaccessible in resource-constrained environments where our target population receives care. The primary innovation of the model lies in its practical utility for diabetic patient stratification, as opposed to biomarker discovery. Our findings indicate that diabetic patients presenting with a concurrent smoking history, elevated total cholesterol levels, and increased lipoprotein(a) concentrations constitute a particularly high-risk subgroup that warrants expedited referral to tertiary care centers equipped with advanced coronary imaging technologies. Moreover, this risk-based triage strategy enables primary care physicians to identify vulnerable diabetic individuals who may benefit from intensified lipid-lowering therapy and smoking cessation interventions while awaiting specialized cardiac assessment. The model’s web-based dynamic nomogram serves as an accessible clinical decision support tool that bridges the gap between basic clinical assessment and advanced imaging. In resource-limited settings where CCTA availability is restricted, this tool enables point-of-care risk stratification using standard laboratory evaluations, potentially reducing unnecessary referrals while ensuring high-risk patients receive appropriate prioritization.

## 5 Limitations

Firstly, this study is an observational clinical study conducted based on our hospital’s cardiology department, with a relatively limited sample size. Therefore, the predictive model still needs to be further validated for its accuracy and clinical application effectiveness in multiple centers with large samples. Secondly, due to the observational clinical study nature of this study, potential confounding factors may not have been fully excluded. Thirdly, although the predictive model’s efficacy was tested through internal validation methods in this study, its effectiveness should be further validated through external validation of the dataset. Fourth, our study did not include assessment of clinical outcomes or long-term follow-up data, which limits our ability to demonstrate the clinical significance of the identified high-risk plaques. Fifth, our study’s approach to endocrine factors was overly simplistic, treating diabetes merely as a binary predictor while neglecting the complex endocrine mechanisms underlying plaque vulnerability. Sixth, our study failed to account for medications with direct plaque-stabilizing effects that could confound the relationship between predictors and plaque vulnerability. Finally, the limited ethnic diversity in our study population means that our prediction model may not perform equally well across different ethnic groups, and the identified predictors may have different relative importance in diverse populations.

## 6 Conclusions

This study developed a diagnostic scoring model for identifying high-risk plaques in patients with new onset UAP by incorporating readily accessible lipid biomarkers alongside traditional risk factors, such as diabetes mellitus and current smoking status. The application of this scoring model enables the preliminary screening of patients with associated high-risk plaques, thereby facilitating the tailored adjustment of clinical treatment strategies for individuals with UAP.

## Data Availability

Individual participant data that underlie the results reported in this article, after de-identification can be obtained from the corresponding author upon reasonable request.
