# Supplementary material for: Developing a novel diagnostic model for identifying high-risk plaques in new onset unstable angina pectoris using coronary CT angiography
Source: Front Endocrinol (Lausanne). 2025 Jul 31;16:1632355. doi: 10.3389/fendo.2025.1632355 (PMC12350121; doi:10.3389/fendo.2025.1632355)

**Supplemental Figure 1. ROC curves of the 5-fold cross-validation.**


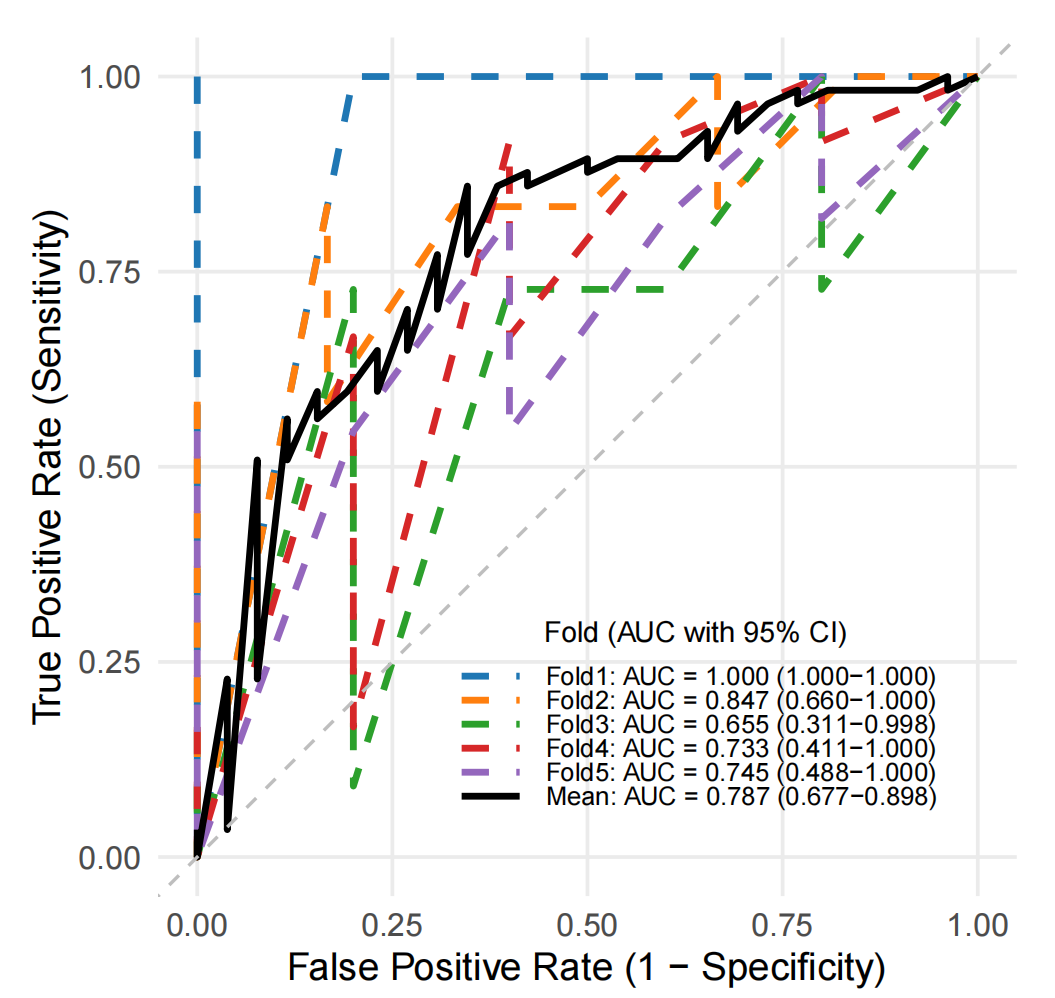


**Supplemental Figure 2. The comprehensive diagnostic performance heatmap demonstrates our model's clinical utility across multiple evaluation criteria.**


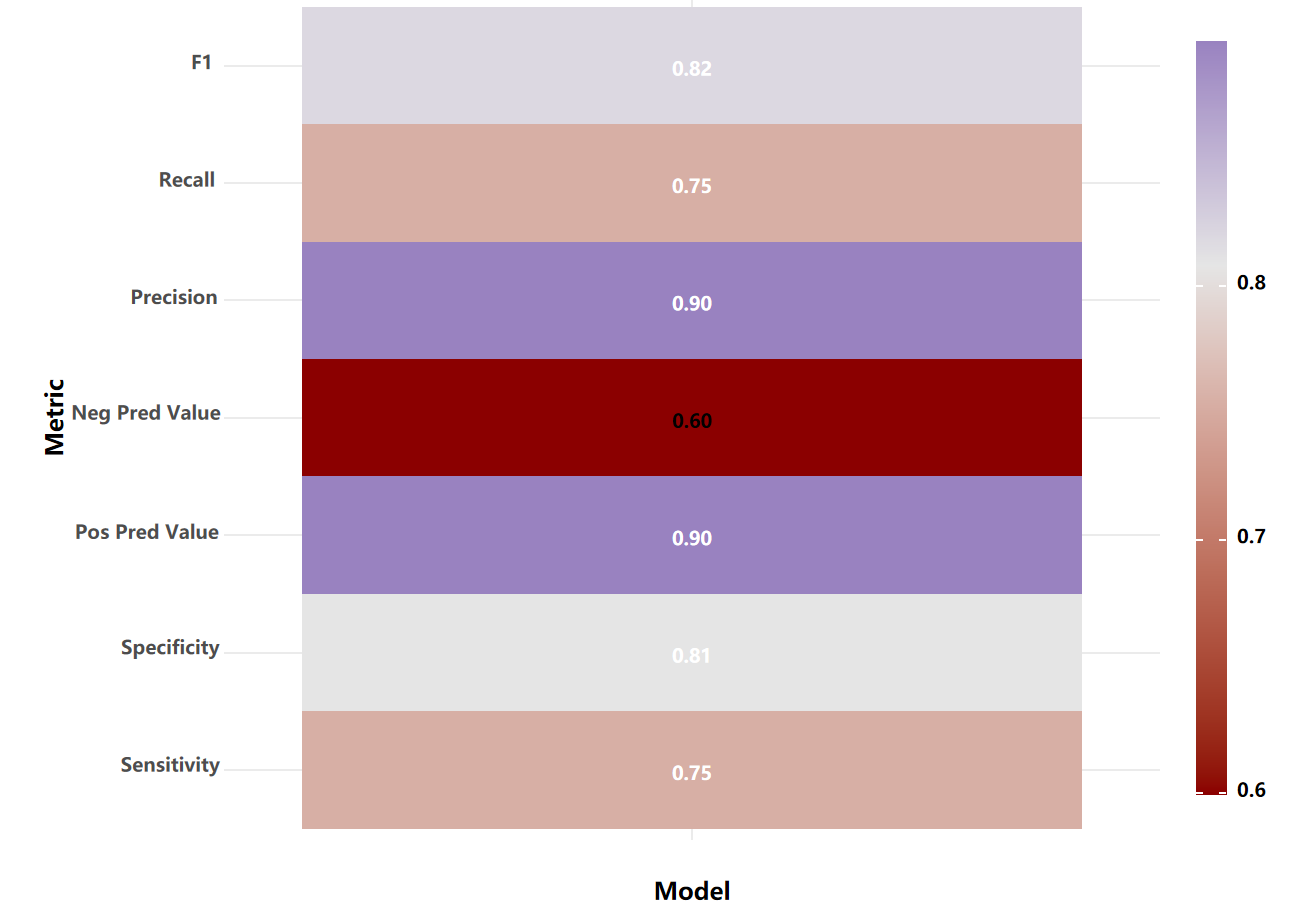

Supplement: Supplementary file 1 [file DataSheet1.docx]
